# Increased morbidity of obese mice infected with mouse-adapted SARS-CoV-2

**DOI:** 10.1038/s41421-021-00305-x

**Published:** 2021-08-25

**Authors:** Ya-Nan Zhang, Zhe-Rui Zhang, Hong-Qing Zhang, Xiao-Dan Li, Jia-Qi Li, Qiu-Yan Zhang, Jing Liu, Qi Li, Cheng-Lin Deng, Zheng-Li Shi, Zhi-Ming Yuan, Han-Qing Ye, Bo Zhang

**Affiliations:** 1 Key Laboratory of Special Pathogens and Biosafety, Center for Emerging Infectious Diseases, Wuhan Institute of Virology, Chinese Academy of Sciences, Wuhan, China; 2 University of Chinese Academy of Sciences, Beijing, China; 3 School of Medicine, Hunan Normal University, Changsha, China; 4 Drug Discovery Center for Infectious Diseases, Nankai University, Tianjin, China

**Keywords:** Mechanisms of disease, Cell signalling

Dear Editor,

Since the outbreak in later 2019, the cofirmed COVID-19 cases have surpassed 162 million, with more than 3 million deaths around the world (https://covid19.who.int), inflicting huge impacts on human health and unprecedented shocks to the global economy. The symptoms of COVID-19 can range from asymptomatic/mild to severe depending on the medical conditions of patients. Numerous studies have suggested that obesity and multiple obesity-associated comorbidities are high-risk factors for severe COVID-19^1,2^. For instance, a meta-analysis about their linkage indicated that people with obesity who contracted SARS-CoV-2 were 74% more likely to be admitted to an ICU, and 48% more likely to die than people of healthy weight^3^. At the same time, based on data from WHO, globally, about 13% of adults aged ≥18 years had obese in 2016, and the rate is still rising. Confronting the double threat of a viral and obesity pandemic, it is thus important to understand how obesity heightens the risk of severe COVID-19 symptoms. To develop a mouse model that is able to mimic obesity-associated COVID-19 diseases is an essential prerequisite for the mechanism investigation as well as the therapeutic evaluation of COVID-19 vaccines and antivirals for the obese population.

COVID-19 modeling in mice has been limited by the species differences in entry receptor of SARS-CoV-2, angiotensin converting enzyme 2 protein, between mice and humans^4^. Using mouse-adapted SARS-CoV-2 is one of efficient solutions to this challenge. In this study, we first obtained a mouse-adapted SARS-CoV-2 (named MP7) through passaging the clinically isolated SARS-CoV-2 strain (WIV04)^3^ in the lungs of old BALB/c mice (9-month-old) for 7 rounds (Supplementary Fig. S1a–c). MP7 produced growth curves similar to those of the parental WIV04 virus in Vero-E6 cells at a multiplicity of infection of 0.01 (Supplementary Fig. S1d), albeit with relatively smaller plaques (Supplementary Fig. S1e). In contrast to poor replication of WIV04, MP7 replicated productively in BALB/c mice upon intranasal inoculation with equal amount of viruses (10^5^ PFU) characterized by much higher copy numbers of viral RNAs in tissues expecially in lungs, nasal turbinates, and trachea (Supplementary Fig. S2b). Moreover, infection of 10^3^–10^5^ PFU of MP7 caused 80%–100% mortality rate in old BALB/c mice within the entire experimental period (Supplementary Fig. S2a). Although MP7 was not lethal for young BALB/c mice (8-week-old) (Supplementary Fig. S2c), apparent weight loss (Supplementary Fig. S2d) and efficient viral replication in the lung of infected mice were observed. Complete genome sequencing of MP7 revealed seven specific nucleotide changes that resulted in six nonsynonymous mutations, including two residue substitutions (Q498H and H655Y) in viral spike (S) protein (Supplementary Table S1) that are the mutations emerging frequently in mouse-adapted strains^5^ or during natural SARS-CoV-2 evolution in humans^6^. It is unclear regarding which mutation(s) may contribute to the lethal disease in old BALB/c mice, as there is no common in mutation profiles between the current MP7 strain and the previsouly reported MA10^7^, to our knowledge, that is the only one lethal mouse-adapted strain of SARS-CoV-2 so far.

Then, the association between obesity and the outcomes of COVID-19 was investigated in MP7-based mouse model. Eight-week-old female C57BL/KsJ-*db/db* mice (a well-estalished mouse model of obesity caused by dysfunctional leptin receptors) and the control C57BL/KsJ-*db/*+ mice were intranasally inoculated with 10^5^ PFU of MP7. Mice from both groups all survived MP7 infection, but the *db/db* mice developed more severe symptoms than the *db/*+ mice, including (i) obvious ruffled fur from 2 to 4 days post infection (dpi), (ii) up to 10% loss of body weight at 4 dpi followed by consisitently low levels of weight till 12 dpi (Fig. 1a), and (iii) massive pulmonary hemorrhage with more macrophage proliferation (yellow arrow), large area of alveolar septal thickening (blue arrow) and infiltration of acute and chronic inflammatory cells near the blood vessels (red arrow) observed on lung sections at 3 dpi by histopathological analysis (Fig. 1b). In contrast, the *db/*+ mice only experienced about 5% reduction in body weight during the first 2 dpi, and thereafter recovered gradually to the original level (Fig. 1a). In addition, much less lung lesions were observed in *db/*+ mice at either experimental time point (Fig. 1b and Supplementary Fig. S3a).

**Fig. 1:**
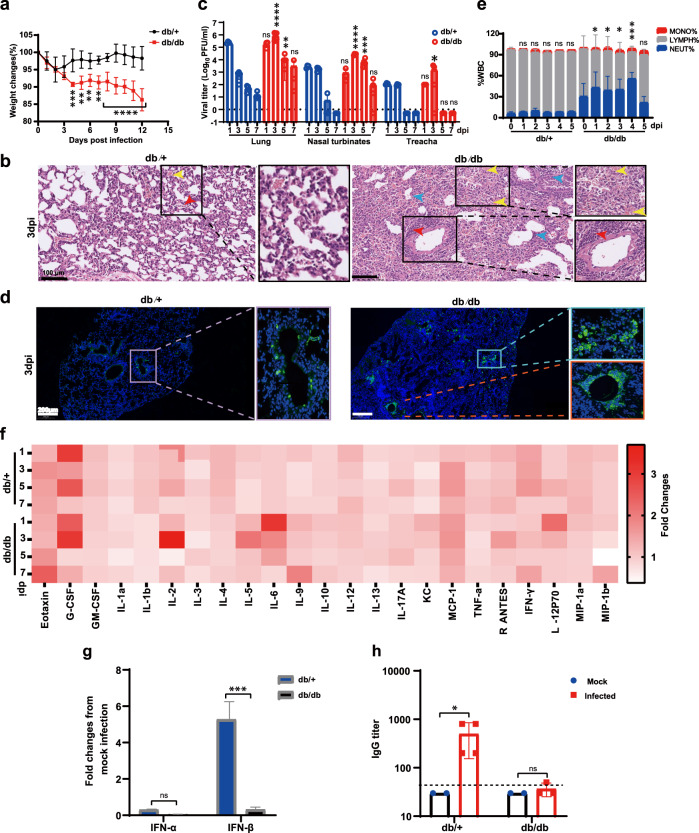
Increased morbidity of obese mice infected with mouse-adapted SARS-CoV-2. Groups of 8-week-old female *db/db* and *db/*+ mice were respectively i.n infected with 10^5^ PFU of MP7 SARS-CoV-2 viruses in a total volume of 50 μL. Infected *db/db* and *db/*+ mice were compared in **a**, weight changes during MP7 infection. Statistical analysis was performed by two-way ANOVA. **P < 0.01, ***P < 0.001, ****P < 0.0001. **b** Representative H&E staining images from groups of mice at 3 dpi. Yellow, red, blue, and black arrows indicate bleeding, inflammatory cell infiltration, alveolar septal thickening, and pulmonary edema, respectively. Scale bars were 100 μm. **c** Viral replication in respiratory tract tissues including nasal turbinates, tracheas, and lungs at 1, 3, 5, 7 dpi. Viral loads in tissues were compared between *db/db* and *db*/+ mice at indicated time points for statistical analysis using the Mann–Whitney U-test. *P < 0.5, ***P < 0.001, ****P < 0.0001, ns not significant. **d** The expression of NP antigen in the lungs was detected by IFA at 3 dpi using anti-NP antibody, and nuclei were stained with DAPI, images were collected using a Pannoramic MIDI system and FV1200 confocal microscopy. Scale bar represents 200 μm. **e** White blood cells (WBC) analysis in the peripheral blood of the mice from 0 to 5 dpi. Statistical analysis in *db/db* or *db*/+ mice was performed using *t*-test compared to the corresponding day 0. *P < 0.5, ***P < 0.001, ns not significant. **f** Serum cytokine/chemokine heatmap in MP7 infected *db/*+ and *db/db* mice. **g** Fold changes of IFN-α and IFN-β mRNA levels relative to mock infection in lung homogenates between *db/*+ and *db/db* mice at 1 dpi. **h** Specific antibody titers of sera from *db/*+ and *db/db* mice at 7 dpi detected by ELISA assay. *P < 0.5, ns not significant. The samples were collected from three mice per group at 1 dpi and four mice per group at 3, 5, 7 dpi. All data are expressed as mean ± standard deviation (SD). Dashed line indicates the detection limit. The above experiments were conducted twice and similar results were obtained. The representative data of one experiment are shown.

At the same time, the viral loads in lungs, nasal turbinates, and trachea were compared between *db/db* and *db/*+ mice at 1, 3, 5, and 7 dpi through plaque assays (Fig. 1c). MP7 could propagate effeciently in both groups of mice, and about 1–3 log higher levels of viral titers, with some exceptions at early or late time points, were detected in the lungs, nasal turbinates, and trachea of *db/db* mice than those of *db/*+ mice. Consistantly, higher levels of viral antigens were observed in the lungs of *db/db* mice compared with *db/*+ mice through immunofluorescence staining with a polyclonal antibody against viral NP protein (Fig. 1d). Notably, in contrast to a sharp decline following peak viral load in *db/*+ mice, *db/db* mice maintained viral load at a relatively high level for an extended period of time. Such differences of either viral load or its diminished rate between control and obese mice may account for different outcomes of COVID-19 oberved in them.

To further assess clinical obesity-associated severe COVID-19 outcomes on mouse model, the neutrophils–lymphocyte ratios (NLRs) in peripheral blood of both groups of mice were measured longitudinally every day from 0 to 5 dpi. Compared with *db/*+ mice, *db/db* mice not only had a relatively higher baseline NLR due to obesity-caused chronic inflammation^8^, but also yielded more enhancement of NLR upon infection before declining and recovering to baseline level at 5 dpi (Fig. 1e), which is consistent with the manifestations of severe patients. In addition, immunohistochemical staining results also showed that more clustering of neutrophils infiltrated into the lungs of *db/db* mice rather than the *db/*+ mice at 3 dpi while large numbers of macrophages infiltrated into the lungs of the both mice (Supplementary Fig. S3b). Meanwhile, 23 cytokines/chemokines in sera were quantified using a Luminex cytokine analysis at different time points. It showed that SARS-CoV-2 infection induced temporal changes in cytokines/chemokines profiles, for instance, Eotaxin, G-CSF, IL-2, IL-5, IL-6, IFN-γ, IL-9, and MCP-1 expressions were increased in *db/*+ and *db/db* mice (Fig. 1f). Notably, IL-6, a major inflammatory indicator implicated with the severity of COVID-19^9,10^, was highly elevated in *db/db* mice upon infection followed by gradual decline, but always much higher than that of *db/*+ mice (except for at the late of infection, 7 dpi). This result also lends support to IL-6 blockade as a feasible therapeutic agent to treat obese patients infected with SARS-CoV-2. We then measured IFN-α and IFN-β mRNA levels in the lungs of infected mice at 1 dpi by qRT-PCR assay (Fig. 1g). It showed that upon infection, (i) IFN-α was not increased in both *db/db* and *db/*+ mice and there was no significant differences between them; (ii) unlike *db/*+ mice whose IFN-β mRNA was upregulated, the expression IFN-β mRNA in *db/db* mice was inhibited (about 0.33-fold relative to mock infection), supporting that severe and critical patients always correlated with impaired type I interferon response^9^.

Moreover, we compared antibody responses to SARS-CoV-2 S protein between both groups (Fig. 1h) using ELISA. A much poorer antibody response was observed in *db/db* mice despite higher viral replication in the respiratory tract, arguing the necessity to weigh COVID-19 vaccine efficacies in obese people.

Overall, our study provides a mouse model demonstrating obesity-associated COVID-19 comorbidities using a mouse-adapted SARS-CoV-2 strain, MP7. It will help elucidate mechanisms of pathogenesis that may be occurring in humans with obesity and accelerate the development of therapeutics for this highly susceptible population, although a longer-term outcome evaluation may be expected to provide more comprehensive information.

## Supplementary information

Supplementary Figures and Tables

## Data Availability

All data are available in the main text or the supplementary materials.
