# Supplementary material for: Increased morbidity of obese mice infected with mouse-adapted SARS-CoV-2
Source: Cell Discov. 2021 Aug 25;7:74. doi: 10.1038/s41421-021-00305-x (PMC8386345; doi:10.1038/s41421-021-00305-x)
Supplement: Supplementary file 1 — Supplementary Figures and Tables [file 41421_2021_305_MOESM1_ESM.pdf]

Supplementary information

Materials and Methods

Ethics statement

BALB/c mice at age of 9 months and 8-10 weeks were provided by the Animal Centre of Wuhan Institute of Virology. 8-week-old C57BL/KsJ-*db/+* and C57BL/KsJ-*db/db* mice were purchased from Changzhou Cavens Laboratory Animal Co., Ltd. Animal studies were carried out in strict accordance with the recommendations in the Guide for the Care and Use of Laboratory Animals. All experiments involving viral infections were conducted in Biosafety Level 3 laboratory (BSL-3) at Wuhan Institute of Virology under a protocol approved by the Laboratory Animal Ethics Committee of Wuhan Institute of Virology, Chinese Academy of Sciences (Permit number: WIVA26201701).

Viruses, cells and antibodies

SARS-CoV-2 strain (WIV04) was originally isolated from a COVID-19 patient. Strain MP7 was developed in this study. SARS-CoV-2 was amplified and titrated by standard plaque forming assay on Vero-E6 cells. Vero-E6 cells were cultured in Dulbecco's modified Eagle's medium (DMEM) containing 10% fetal bovine serum (FBS), 100 units/mL of penicillin and 100 μg/mL of streptomycin and maintained in 5% CO_2 at 37 °C. Rabbit anti-RP3-CoV N protein polyclonal antibody was kindly provided by Prof. Bing Yan at Wuhan Institute of Virology.

Serial passage of SARS-CoV-2 in aged BALB/c mice.

Adaptation of SARS-CoV-2 was achieved by serial passage of lung homogenates of infected BALB/c mice. A dose of 1×10^5 plaque forming unit (PFU) of SARS-CoV-2 was administered intranasally (i.n.) to three anesthetized, female 9-month-old BALB/c mice in a total volume of 50 μL. Three days after inoculation, the mice were euthanized, and the lung of each mouse was removed and homogenized as a 10% w/v suspension in DMEM. The lung homogenate was clarified by centrifugation at 13, 000 rpm for 5 min, and the supernatants of lung homogenates from three mice were administered i.n. into three naïve mice after filtration and mix completely. The process of i.n. inoculation of 3 female aged BALB/c mice was repeated for 7 times. After detection of SARS-CoV-

2 RNA in lung homogenates, viral load was estimated by plaque assay. Virus from passage 7, named MP7, was amplified once in Vero-E6 cells and aliquoted for the following studies.

Measurement of viral RNA.

Tissue homogenates were clarified by centrifugation at 13, 000 rpm for 5 min, and the supernatants were transferred to a new EP tube. And viral RNA was extracted using QIAamp viral RNA mini kit（52906，Qiagen）following the manufacturer's protocol. qRT-PCR assay was performed using Luna® Universal Probe One-Step RT-PCR Kit (E3006). The primers and probe based on the SARS-CoV-2 S gene were designed as:

RBD-qF1: 5'- CAATGGTTTAACAGGCACAGG-3'

RBD-qR1: 5'- CTCAAGTGTCTGTGGATCACG-3'

Probe: ACAGCATCAGTAGTGTCAGCAATGTCTC

Enzyme linked immunosorbent assay (ELISA)

SARS-CoV-2 antibody titer of serum samples from immunized mice was determined by indirect ELISA assay. 96-well microtiter plates were coated with 2 μg purified receptor binding domain (RBD) of spike protein for each well at 2-8°C overnight, and blocked with 5% skim milk for 1 h at room temperature. Diluted sera were applied to each well and incubated for 2 h at 37°C, followed by incubation with goat anti-mouse antibodies conjugated with HRP for another 1 h at 37°C after 3 times PBS wash. Following addition of 1 M H_2SO_4 to stop the reaction, the plate was developed using TMB and read at 450 nm by ELISA plate reader for final data.

Histological Analysis

Lung samples from mice were fixed with 4% paraformaldehyde, embedded in paraffin followed by sagittal sections at 4-μm thickness on a microtome, and mounted on APS-coated slides. For histopathological analysis, sagittal sections were directly stained with H&E. For detection of SARS-CoV-2 antigen in fixed lungs, indirect immunofluorescence assay (IFA) was conducted. Briefly, the slides were deparaffinized, rehydrated and experienced heat-induced antigen retrieval with EDTA (pH 8.0) in a microwave oven. Then tissues were uniformly covered with 5% BSA for incubation at room temperature for 1 h followed by addition of primary antibody (anti-RP3-CoV N

protein polyclonal antibody, 1:500) and PBS wash. After the slices were slightly dried, tissues were incubated with 488s-conjugated goat-anti-rabbit IgG (Abcam, GB25301) at 1:200 dilution for 40 min. After washing in PBS, slides were stained with DAPI (Beyotime) at 1:100 dilution. The image information was collected using a Pannoramic MIDI system (3DHISTECH, Budapest) and FV1200 confocal microscopy (Olympus).

Blood sampling and biochemistry

Blood was collected retro-orbitally and transferred to a blood collection tube containing EDTA to prevent clotting. Cell classification was analyzed by a ProCyte Dx Hematology Analyzer (IDEXX).

Cytokine and chemokines analysis.

Cytokines and chemokines in mouse sera were measured using Bio-Plex Pro Mouse Cytokine Grp I Panel 23-Plex (Bio-Rad, USA) according to the manufacturer's protocol. The data were collected on Luminex 200 and analyzed by Luminex PONENT (Thermo Fisher, USA).

Cytokine mRNA quantification

To analyze cytokine mRNA levels in infected mouse lungs, 100 μL of lung homogenates were lysed in 1 ml Trizol and RNA was extracted according to manufacturer's protocol followed by qRT-PCR assay. Primers: 5′-ATGGCTAGGCCCTTTGCTTTC-3′ and 5′-CTGTGTACCAGAGGGTGTAGTT-3′ for IFN-α; 5′-CAGCTCCAAGAAAGGACGAAC-3′ and 5′-GGCAGTGTAACTCTTCTGCAT-3′ for IFN-β.

Statistical Analysis

All data were analyzed using GraphPad Prism 8.0.2 software and all results are expressed as mean ± standard deviation (SD). Statistical significance was assigned when P values were < 0.05. Student's t-test was used to determine the differences between two groups.

Supplementary Figures

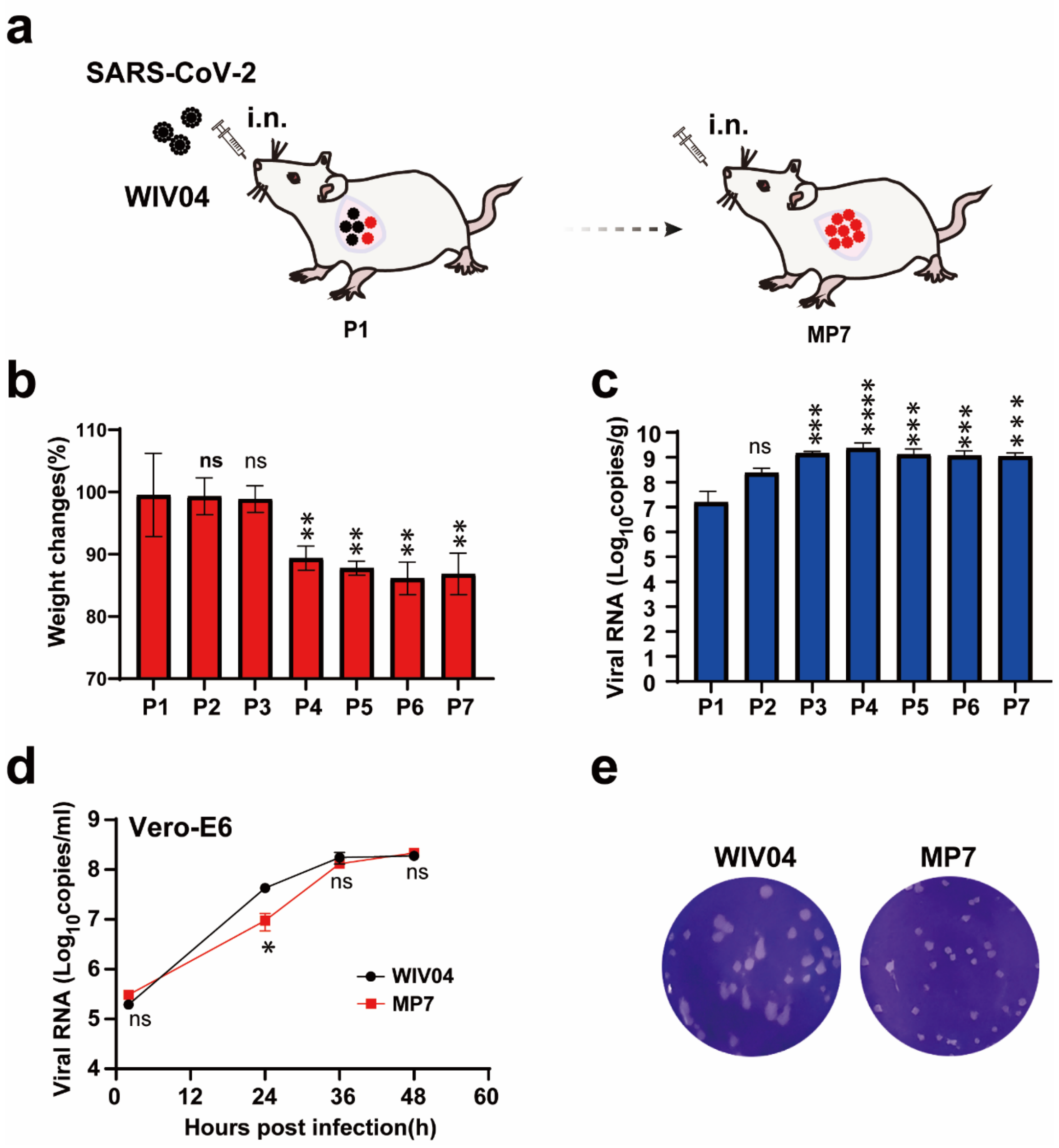

Fig. S1 Generation and characterization of a mouse-adapted strain of SARS-CoV-2 in BALB/c mice. a, Schematic diagram of the passage of SARS-CoV-2 in 9-month - old BALB/c mice. The original SARS-CoV-2 viruses are shown in black, and the adapted viruses are shown in red. b, Weight changes (n=3) of passaged mice from P1 to P7 on day 3 after infection. Weight changes of the indicated passage relative to P1 were shown and statistical analysis was performed using One-way ANOVA. ** P <0.01, ns, not significant. c, Viral loads in mouse lungs (n=3) from P1 to P7 on day 3 after infection characterized by the copy numbers of viral genomic RNA. Viral RNA copies were determined by quantitative reverse transcription polymerase chain reaction (qRT-

PCR) assay. Data are presented as means ± SD (n = 3 mice per passage). Viral RNA loads of the indicated passage relative to P1 were shown and statistical analysis was performed using One-way ANOVA. *** P <0.001, ****P <0.0001, ns, not significant. d, Comparison of growth kinetics between WIV04 and MP7 viruses in Vero-E6 cells. Vero-E6 cells were infected with WIV04 or MP7 viruses at an MOI of 0.01. At the indicated times, supernatants were collected and viral titers were measured by qRT-PCR assays. Statistical analysis was performed by Two-way ANOVA. *P <0.5, ns, not significant. e, Plaque morphology of WIV04 and MP7 viruses on Vero-E6 cells.

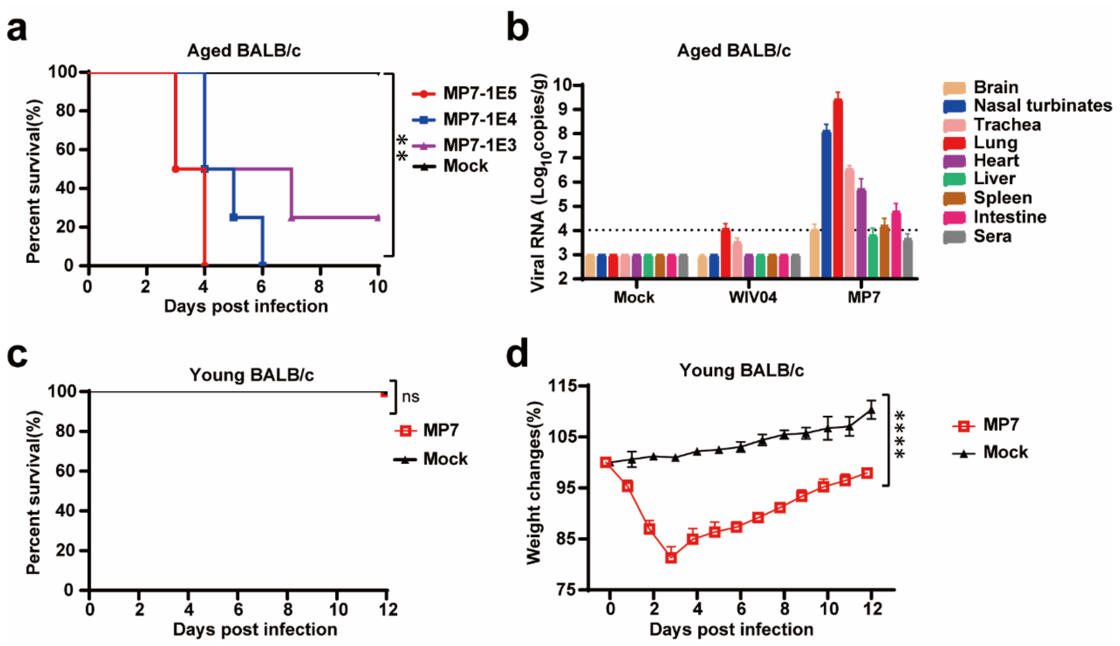

Fig. S2 SARS-CoV-2 MP7 was lethal in aged BALB/c mice. a, Survival curve of MP7 in aged mice. 9-month-old female BALB/c mice (n = 4 per group) were infected with 10^3 , 10^4, or 10^5 PFU of MP7 and mock-infected mice were included as the negative control. b, Tissue distribution of viruses in MP7 infected aged mice. 9-month-old female BALB/c mice (n = 4 per group) was infected with 10^5 PFU of MP7 and WIV04, respectively. And mock-infected mice were used as the negative control. At 3 dpi when the mice infected with MP7 were on brink of death, the tissues were collected and subjected to qRT-PCR assay for quantification of viral RNA. c-d, Survival (c) and weight changes (d) in young BALB/c mice infected with MP7 during 12 days after infection. 8- to10-week old female BALB/c mice (n = 4 per group) were infected with

10^5 PFU of MP7 or PBS (mock). Log-rank test was used for statistical analysis of survival and Two-way ANOVA was used for statistical analysis of weight changes. **P <0.01, ****P <0.0001, ns, not significant.

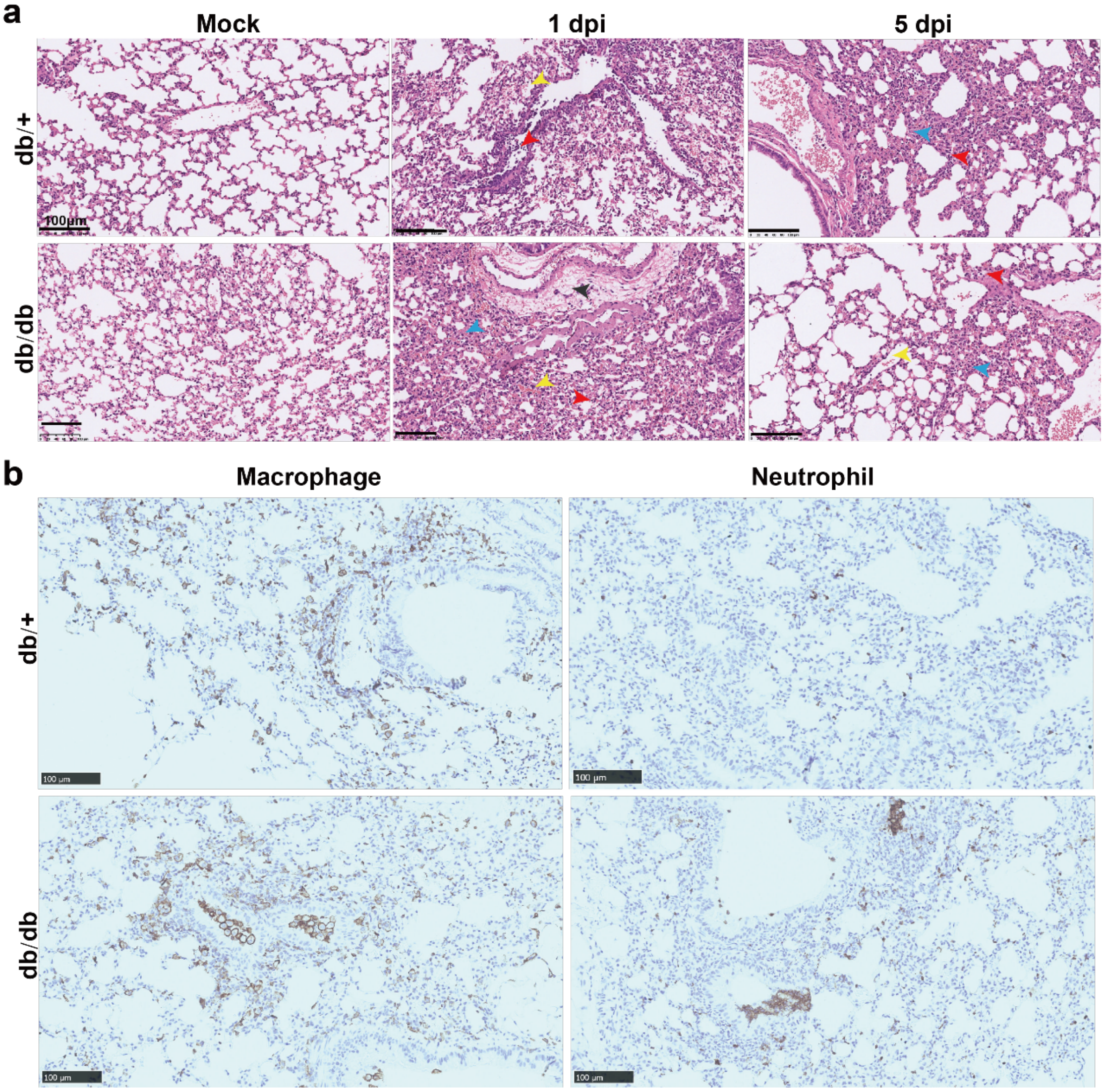

Fig. S3 Pathological changes of SARS-CoV-2 MP7 in db/db mice.

a. Representative H&E staining images from groups of mice at 1,5 dpi. Yellow, red, blue and black arrows indicate bleeding, inflammatory cell infiltration, alveolar septal thickening and pulmonary edema, respectively. b. Immunohistochemical staining of macrophage and neutrophil cells of lungs in db/db and control mice at 3 dpi when the lung pathology was most severe. All scale bars present 100 μm.

Supplementary Tables

Table S1. Genomic changes in MP7 SARS-CoV-2 compared with WT SARS-CoV-2 (WIV04).

Nucleotide	WIV04	MP7	Amino acid change	Amino acid position
3504	A	C	Asn→Thr	nsP3-262
12147	A	G	Gln→Arg	nsP8-19
12884	A	G/A	Thr→Ala	nsP9-67
17181	T	C	None	nsP13
17321	C	T	Ala→Val	nsP13-362
18844	G	A	Val→Ile	nsP14-269
23056	A	C	Gln→His	S-498
23525	C	T	His→Tyr	S-655
